# Utilization and associated factors of growth monitoring and promotion services among young children in Gorkha district of Nepal

**DOI:** 10.1186/s40795-024-00978-z

**Published:** 2024-12-19

**Authors:** Jagat Prasad Upadhyay, Damaru Prasad Paneru, Yam Prasad Sharma, Nava Raj Khadka

**Affiliations:** 1 School of Health and Allied Sciences, Pokhara University, Pokhara, Nepal; 2 Government of Gandaki Province, Ministry of Health, Health Office Gorkha, Gorkha, Nepal

**Keywords:** Growth monitoring and promotion, Utilization, Children, Gorkha, Nepal

## Abstract

**Introduction:**

Promotion of child health during the first thousand days from conception to the child’s second birthday is vital for survival, growth and development. Growth monitoring and promotion services are key to the early detection of growth faltering and preventing malnutrition and promoting child health. This study aimed to assess the utilization of Growth Monitoring and Promotion (GMP) services and its associated factors among young children in Gorkha district of Nepal.

**Methods:**

A community-based cross-sectional study was conducted in the Gorkha district, involving 290 mother-child pairs, from April 2024 to June 2024. Multistage random sampling was used to select mothers. Data was collected through face-to-face interviews using structured questionnaires. Descriptive statistics and inferential statistics involving multivariate logistic regression analyses were performed to identify the factors associated with GMP service utilization.

**Results:**

Only 5.5% of children had completed the recommended 24 GMP visits according to protocol, while 23.8% utilized GMP services more than 15 times, which represents the 75th percentile of service utilization. Mothers who had good knowledge had significantly higher (adjusted OR = 4.23, 95% CI: 2.070–8.650, *p* < 0.001) GMP service utilization among their children than those counterpart mothers with poor knowledge about the GMP services. The main reasons for not regular utilizing GMP services included the time constraints and their household responsibilities (85%).

**Conclusion:**

This study reported the low utilization of GMP services among children in Gorkha district. Maternal knowledge on GMP service emerged as the primary predictor of GMP service utilization and maternal engagement in the household activities explores as a leading barrier to GMP service utilization. Enhancing maternal knowledge through community engagement strategies and improving the monitoring mechanism within the health systems could increase GMP utilization.

## Introduction

Promotion of child health during the first thousand days starting from conception to child’s second birthday is vital for their survival, growth and development [1]. Growth monitoring and promotion (GMP) is a critical component of child health programs in Nepal [2]. Growth Monitoring and Promotion (GMP) services involve proactive use of growth monitoring (GM) to measure and interpret growth, enhancing communication and engagement with parents. This approach aims to prompt appropriate actions to support child growth by boosting parents/caregivers’ understanding of child growth, enhancing caregiving practices, and increasing demand for necessary services [2, 3]. Monitoring children’s growth ensures timely identification of potential health concerns, enabling early intervention through individual care or population-wide health initiatives and prioritizing child growth promotion holds immense potential for both reducing child mortality and alleviating poverty [4, 5]. Globally, malnutrition remains a serious public health problem. In 2022, an estimated 149 million children under five years were stunted, 45 million were wasted, and 37 million were overweight or obese [6]. GMP is often integrated with other child health services such as immunization and vitamin A supplementation, enhancing its reach and effectiveness. Female Community Health Volunteers (FCHV) play a crucial role in promoting GMP services and conducting community-based monitoring, particularly in rural areas [7]. Municipalities in Nepal have peripheral health outlets, and these peripheral health outlets also operate extension clinics where growth monitoring services are provided. Growth monitoring is also regularly conducted at maternal and child health clinics. Through these efforts, Nepal aims to achieve universal access to healthcare services.

In Nepal, National Nutrition Program is one of the priority programs; however, child malnutrition persists with 25% stunted, 19% underweight and 8% wasted [8]. There is lacking comprehensive research on GMP utilization due to which there is deficiency in evidence-based interventions and policies. Several studies revealed that chronic child malnutrition is highly prevalent in Gorkha district [8, 9]. Government of Nepal recognized the importance of GMP services, and it has made the universal coverage through its health system network; nonetheless, there is a significant gap regarding the status of GMP service utilization and factors influencing GMP service utilization in Nepal including the Gorkha district. This gap persists though the continued efforts made to reduce nutritional problems across the country including Gorkha district [8, 9]. Few studies have comprehensively examined both supply and demand-side barriers of growth monitoring and promotion services in Nepal; however, these evidences lack in the assessment of GMP service utilization and related factors [10]. Meanwhile, Government of Nepal has prioritized addressing malnutrition through the Multi-Sectoral Nutrition Plan (MSNP) reflecting its commitment to achieving multiple Sustainable Development Goals [11]. Effective strategies to improve GMP utilization require contextual evidence on socioeconomic, cultural and service delivery factors. This study aimed to assess the utilization of growth monitoring and promotion services and its associated factors among children aged 24-35 months in Gorkha district of Nepal. Findings from this study could contribute to the development of interventions mechanisms tailored to improve GMP services, thereby contributing to Nepal’s aim to achieve child health-related Sustainable Development Goals.

### Study Design and setting

A community based cross-sectional study was conducted in Gorkha district from April 2024 to June  2024. Gorkha district lies in the Gandaki Province of Nepal. There are eleven local levels, with two urban municipalities and nine rural municipalities. It has an area of 3,610 square kilometers and had a population of 251,057 [12]. Health information system of Nepal reported that there were 4223 children aged 24–35 months in the fiscal year 2022/23. Growth monitoring and promotion (GMP) services in Gorkha district are provided through 2 Government Hospitals, 3 Primary hospitals, 2 Primary Health care centers, 66 Health Posts, 44 Community Health units, 11 Urban Health clinics, 4 Basic Health service centers and 1 Private hospital and several extended outreach clinics. Despite the service existence, prevalence of chronic child malnutrition is high in Gorkha district [9, 13].

## Study participants and sampling

The study population constituted the mother-child pairs with children aged 24–35 months. A multi-stage random sampling was employed to select desired sample participant. In the first stage out of a defined eleven local level administrative unit arranged alphabetically, six were selected using simple random sampling. In the second stage the selected rural/municipalities number of ward from each of the selected local level administrative unit chosen with the ratio of 1:5. Rural municipalities constitute a minimum of eight to maximum nine wards while municipality have minimum of ten to fourteen wards. In the next stage, a list of villages was obtained from the selected wards. Number of households residing in a defined territory classified by the local unit or social groups term the village in this study. Thereafter list of children aged 24–35 months in each of the villages was acquired from public health facilities of selected ward, corresponding the updated list maintained by female health community volunteer for the recent purpose measles rubella immunization campaign. The required sample size of 290 participants was then allocated proportionately to each of the selected ward based on the population of children aged 24–35 months. Finally, study participants were selected from the chosen villages using simple random sampling. Thus, the selected participants were then traced from their residential address and the study team visited their household. All the mother- child pairs with their children aged 24–35 months residing in the selected local level of Gorkha district at least one year prior to the study were included. Mother with child aged of 24–35 months who were seriously ill, unable to communicate and those who did not have Child Health Card were excluded from the study.

### Data collection tools and procedures

Data collection tool was developed based on an extensive literature review and expert consultations. A structured interview schedule was used to collect information from mothers. The questionnaire consisted of four sections: socio-demographic characteristics, information on utilization of maternal and child health services, maternal knowledge and attitudes towards GMP service and utilization of GMP services. Face-to-face interviews were conducted with mothers using pretested interview schedules after obtaining written informed consent.

## Operational definitions

- **Young Children**: Children from birth up to the age of two years are considered young children in this study.
- **Antenatal Care (ANC) Utilization**: Mothers who utilized ANC services for 4 or more times during pregnancy as per protocol.
- **Postnatal Care (PNC) Utilization**: Mothers who utilized PNC services 2 or more times as per protocol were considered PNC utilization.
- **Maternal Knowledge**: Knowledge of mothers toward GMP service utilization are categorized as good knowledge and poor knowledge based on literature and assessed using ten knowledge related questions. Each question has two responses (yes = 1 or 0 = no). Thus, the total score ranges from 0 to 10. A score above 7 was categorized as good knowledge and below 7 was categorized as poor knowledge [14]; aligning with the literatures.
- **Maternal Attitude**: The attitude of the mother to GMP service was assessed by 9 attitude questions using Likert scale measures (1 = Strongly Disagree, 2 = Disagree, 3 = Neutral, 4 = Agree and 5 = Strongly Agree). The total score ranges from 9 to 45. The lowest possible score was 9, and the highest possible score was 45. Score of < 80% was categorized as unfavorable attitude and a score of ≥ 80% was categorized as favorable attitude based on blooms taxonomy of attitude assessment [15].
- **Child Health Card Utilization**: Mothers who utilized Growth Monitoring and Promotion (GMP) services at least 7 times during the visit of immunization and had the visits recorded in their child’s health card were considered to have utilized the child health card. Those who did not meet these criteria were considered non-utilized.
- **GMP Utilization**: Mothers who visited health facilities 24 times to monitor the weight of their children and had the information recorded in the child’s health card were considered as having utilized GMP services per protocol. Nonetheless, the findings revealed that only 5.5% utilized GMP services. For statistical estimation, the utilization was expressed in percentile. Mothers above the 75th percentile were those who utilized GMP more than 15 times, and mothers below the 75th percentile were those who utilized GMP 15 times or fewer.
- **Rural/Urban municipality criteria**: According to the Local Government Operation Act 2017, rural/municipalities in Nepal are classified based on five key criteria: population density, where larger concentrations indicate urban status; geographical coverage, with scattered populations typically categorized as rural; infrastructure development level, requiring urban areas to have established facilities like roads, utilities, schools, and hospitals; economic activities, where urban areas demonstrate higher commercial and industrial engagement compared to agriculture-dominant rural areas.

### Data processing and analysis

The data were entered into EpiData 3.1 and then exported to IBM SPSS (Statistical Package for Social Sciences) version 22 for analysis. Descriptive statistics, such as frequencies, percentages, mean, median and standard deviation were calculated to describe the findings and presented in text and tables. A chi-square test was performed for all variables to check the assumptions and identify differences in the utilization of GMP service. Bivariate analysis was done using logistic regression analysis, and all variables associated with the utilization of GMP services, with statistical significance at a 95% confidence level and *p* < 0.05, were identified. Variables that were significant with the chi-square test were subjected to the multi-collinearity checks. A multicollinearity test was performed using variance inflation factors (VIF), and variables with VIF less than 2 were included in the multivariate model. Furthermore, the goodness of fit for the final logistic model was tested using the Hosmer and Lemeshow test. Both crude and adjusted odds ratios with the corresponding 95% confidence interval, p-value was calculated to measure the strength and presence of association between GMP service utilization with its associated factors.

## Results

### Sociodemographic characteristics of the participants

In Table 1, a total of 290 mothers with children aged 24–35 months were included, yielding a 100% response rate. The mean (± SD) age of the children was 28.84 ± (3.56) months. More than half (55.5%) of the mothers had male children. Less than half (44.1%) of the mothers were from Janajati communities, and nearly three-quarter (72.8%) of the participants were Hindus. Almost three-fifths (59.3%) of the mothers had a household family size of less than five. More than one-third (38.3%) of the mothers had secondary education, followed by basic-level education (32.8%). Almost three-fourths (74.8%) of the mothers were housewife (Table 1).

**Table 1:** Sociodemographic characteristics of the participants

Characteristics	Frequency (*n* = 290)	Percentage (%)
**Age of child (months)**		
≤ 30	194	66.90
> 30	96	33.10
Mean age (± SD)	28.84 ± (3.56)	
**Sex of the child**		
Male	161	55.50
Female	129	44.50
**Ethnicity of mother**		
Brahmin/Chettri	50	17.25
Janajati	128	44.13
Muslim	34	11.72
Dalit	78	26.90
**Religion of mother**		
Hindu	211	72.76
Buddhism	8	2.76
Islam	34	11.72
Christian	37	12.76
**Family size**		
≤ 4	88	30.30
> 4	202	69.70
**Educational status of mother**		
Who cannot read and write	12	4.10
Non formal education	32	11.00
Basic level	95	32.80
Secondary level	111	38.30
Bachelor and above	40	13.80
**Occupational status of mother**		
Housewife	217	74.82
Government Job	13	4.48
Personal business	33	11.38
Farmer	19	6.56
Private Job	8	2.76
**Educational status of Father**		
Who cannot read and write	3	1.00
Non formal education	35	12.10
Basic level	105	36.20
Secondary level	102	35.20
Bachelor level	45	15.50
**Occupational status of Father**		
Agriculture	39	13.40
Business	43	14.80
Government job	37	12.80
Foreign employment	88	30.30
Daily wage/labor	57	19.70
Private job	18	6.20
Unemployment	8	2.80

*Note* Hill Janajati groups: Magar, Gurung, Rai, Limbu, Sherpa, Sunuwar, Bhote, Raji, Raute and others

Terai Janajati groups: Tharu, Dhimal, Gangain, Satar/Santhal, Dahngar/Jhangar, Koche, Meche and others

Low caste Hindu groups or Dalits: Kami, Sarki, Damai, Badi and Gaine [16]

### Maternal and Child Health Service utilization

Almost all mothers (97.9%) delivered their latest child at a health institution, and the majority (82.1%) of them delivered their children through normal delivery. 100% of the mothers had at least one ANC visit. More than half of the mothers (56.7%) had ≤ 4 ANC visits and < 2 PNC visits (54.8%) as per the protocol. The majority (90%) of the participants lived within five kilometers or less from a health facility. Most of the participants (89%) waited 30 min or less at the health facility to receive child health services. All mothers receive counseling on GMP service and utilize child health card (Table 2).

**Table 2:** Maternal and Child Health Service utilization

Characteristics	Frequency (*n* = 290)	Percentage (%)
**Place of delivery**		
Health institution	284	97.90
Home	6	2.10
**Mode of delivery**		
Normal delivery	238	82.10
Cesarean section	52	17.90
**Child immunization status**		
Full immunized	290	100
**Had ANC Check up**		
Yes (at least one)	290	100
**Frequency of ANC visit (times)**		
≤ 4	165	56.90
> 4	125	43.10
**Frequency of PNC visit (times)**		
< 2	159	54.80
≥ 2	131	45.20
**Distance from Home to health facility (HF)**		
≤ 5 km	261	90.00
> 5 km	29	10.00
**Waiting time at HF to get GMP services**		
≤ 30 min	258	89.00
> 30 min	32	11.00
**Counseling on GMP Service**		
Yes	290	100
**Utilization of child health card**		
Yes	290	100

### Maternal knowledge and attitude toward GMP services

This study revealed that 49% and 85.9% of mothers have good knowledge about the GMP services and favorable attitude towards GMP services respectively (Table 3).

**Table 3:** Maternal knowledge and attitude toward GMP services

Characteristics	Frequency (*n* = 290)	Percentage (%)
**Maternal knowledge toward GMP services**		
Good Knowledge	142	49.00
Poor Knowledge	148	51.00
**Attitude of Mother toward GMP services**		
Favorable attitude	249	85.90
Unfavorable attitude	41	14.10

### Growth monitoring and promotion service utilization

All mother-child pairs used GMP services at least once; however, unexpectedly, a very low proportion of them (5.5%) utilized the complete GMP services as per protocol. Similarly, less than a quarter (23.8%) of them used > 15 times (75th percentile) the GMP services. Majority of the respondents (85.5%) mentioned that the time constraints and household responsibilities as the primary reasons for not utilizing regular GMP services (Table 4).

**Table 4:** Growth monitoring and promotion service utilization

Characteristics	Frequency	Percentage (%)
**Use of GMP service**		
Used GMP service at least one time	290	100
**Utilization of complete GMP (as per protocol)**		
< 12 times	140	48.30
13 to 23 times	134	46.20
24 times	16	5.50
**Utilization of GMP service**		
Utilized > 15 times (75th percentile)	69	23.80
Utilized ≤ 15 times	221	76.20
Reason for not utilizing regular GMP service*		
Time Constraints and household responsibility	248	85.51
Health facility is far	21	7.24
Not felt necessary as my child is healthy	19	6.56
Unaware about GMP service	20	6.90
Forgot fullness	10	3.44

*Multiple responses

### Factors Associated with the utilization of GMP Service

Factors that were statistically significant with GMP service utilization using Chi-square test were subjected for bivariate and multivariate regression analysis. Sex of child, ethnicity of mother, religion, educational status of mother and father, occupation of father, waiting time at health facility, knowledge and attitude of mother toward GMP services were identified as the significant factors associated with GMP service utilization, however age of child, family size, occupation of mother, place of delivery, mode of delivery, frequency of ANC visit, frequency of PNC visit, distance from health facility to home were not significantly associated with GMP service utilization in chi-square test. After controlling potential confounding, only maternal knowledge of GMP service was significantly associated with GMP service utilization in multivariate regression analysis. The mothers with good knowledge about GMP services were 4.23 (95% CI: 2.070–8.650, *p* < 0.001) times more likely to utilize GMP services than those mothers who have poor knowledge (Table 5).

**Table 5:** Factors Associated with the utilization of GMP Service

Variables	Crude OR (95% CI)	*p*-value	Adjusted OR (95% CI)	*p*-value
**Sex of Child**				
Male	1.84 (1.047–3.246)	0.034*	1.55 (0.15–2.956)	0.181
Female	1		1	
**Ethnicity of Mother**				
Janajati	2.43 (1.247–4.748)	0.009*	1.85 (0.861–3.996)	0114
Brahmin/Chettri	3.96 (1.801–8.720)	0.001*	1.69 (0.625–4.558)	0.302
Muslim and Dalit	1		1	
**Religion of Mother**				
Hindu	3.09 (1.451–6.580)	0.003*	2.02 (0.851–4.831)	0.111
Non-Hindu	1		1	
**Education status of Mother**				
Basic level and below	1		1	
Secondary level	2.97 (1.567–5.622)	0.001*	1.40 (0.545–3.587)	0.485
Bachelor and above	4.97 (2.235–11.045)	< 0.001*	0.76 (0.196–2.905)	0.683
**Husband Educational status**				
Basic and non-formal education	1		1	
Secondary level	3.63 (1.862–7.072)	< 0.001*	2.29 (0.860–6.088)	0.97
Bachelor and above	6.95 (3.174–15.199)	< 0.001*	3.19 (0.816–12.506)	0.95
**Husband Occupational Status**				
Agriculture	1.61 (0.625–4.152)	0.323	1.36 (0.463–3.977)	0.578
Business	2.29 (0.963–5.456)	0.061	0.81 (0.272–2.411)	0.705
Government service	3.58 (1.395–9.193)	0.008*	1.18 (0.348–4.029)	0.787
Foreign Employment	0.67 (0.265–1.702)	0.401	0.44 (0.152–1.272)	0.129
Daily Wages/labor	1		1	
**Waiting time at HF to get GMP services**				
≤ 30 min	11.09 (1.486–82.844)	0.019*	6.31 (0.727–54.720)	0.095
> 30 min	1		1	
**Maternal Knowledge on GMP**				
Good Knowledge	4.92 (2.642–9.136)	< 0.001*	4.23 (2.070–8.650)	< 0.001*
Poor Knowledge	1		1	

*Statistically significant at *p* < 0.05, 1 = reference category

## Discussion

The study assessed the utilization of growth monitoring and promotion services and its associated factors among children aged 24–35 months in Gorkha districts of Nepal. This study revealed very low (5.5%) GMP service utilization as per protocol (24 times visit under two years) in the study area. This extremely low level of utilization is a key concern, and the poor attention was given on GMP services despite its indispensable role for tracking child growth, early identification of nutritional issues and promoting healthy child development. To enable the statistical calculation, GMP utilization was categorized using the cutoff 75th percentile: Mothers above the 75th percentile was those who utilized GMP services more than 15 times, and mothers below the 75th percentile were those who utilized GMP services 15 times or fewer. Mothers who utilized GMP services for their children > 15 times were only 23.8%. Similar studies from different countries constituted the samples of mother-child pair of 0–23 months, however In the present study mothers of children aged 24–35 months have been interviewed and the utilization of GMP services has been assessed for the age group less than 24 months [17].

GMP service utilization in this study was lower than those reported in similar studies. These studies reported the minimum of 13.7% GMP service utilization in Northwest Ethiopia and the highest utilization (87%) in Afghanistan [12, 14–16, 18–25] (11–15) [19–25]. Low utilization of GMP services in Nepal might be due to geographical barriers, lack of infrastructure and limited health care resources particularly in rural areas which limits its accessibility. Countries with stronger economy might have more resources to provide healthcare professionals, infrastructure, and transportation to their citizens; leading to increase the GMP service utilization [22]. The inconsistency could be attributed to differences in the socioeconomic characteristics of the study populations, study designs, and study periods [18, 24, 26]. Additionally, this study further revealed that 85.5% of mothers do not utilize the GMP services due to time constraints and household responsibilities. This highlights the mother’s primary engagements in household responsibilities placing the GMP service utilization as secondary. It demands the mother’s priority on child health care in terms of time and the need for interventions to address these barriers.

Maternal knowledge remains a significant factor associated with utilization of GMP services, while other factors did not show significant association at the multivariate analysis. Mother who has good knowledge on growth monitoring and promotion services were 4.23 times more likely to utilize GMP service compared to those mothers with poor knowledge. This finding is supported by the study conducted in several part of Ethiopia [18, 22, 23, 25, 27–29]. The possible explanation for this is mothers with adequate knowledge about growth monitoring and promotion (GMP) programs are more likely to understand the information displayed on growth charts. This understanding motivates them to utilize GMP sessions. Additionally, mothers who have good knowledge about GMP are aware of the potential adverse outcomes of not utilizing these services, leading them to actively engage in GMP. The presence of maternal knowledge regarding GMP contributes to improved nutritional status and growth in children. It also enhances infant and young child feeding practices, as mothers become better informed about proper nutrition and care practices [22, 23, 28]. This indicates that improving mothers’ awareness and understanding of GMP is likely crucial for increasing service utilization. The study also highlights the critical need for specific strategies to enhance maternal knowledge and community awareness about the significance of GMP service for child growth and development. Interestingly, though 49% of mothers demonstrated good knowledge about GMP services, only 23.8% utilized the services more than 15 times. This discrepancy suggests that knowledge alone may not be sufficient to ensure utilization and other barriers such as time constraints and accessibility issues need to be addressed concurrently.

The study has several strengths, including its community-based approach and high response rate. However, limitations include potential recall bias as mothers were asked about service utilization over a two-year period. The study focused on a specific geographic region, potentially limiting the generalizability of the findings to other contexts. Selection bias was also a concern, as only mothers who possessed child health cards were included in the research sample. Furthermore, different methodological approaches, sample sizes, and data collection techniques employed by researchers could have led to different findings.

## Conclusion

The utilization of GMP services is very low as only 5.5% utilize GMP services 24 times as per protocol until child second birthday. While considering the 75th percentile 23.8% utilized GMP services more than 15 times. Maternal knowledge on GMP emerged as the primary predictor of GMP service utilization. Majority of the mother cited time constraints and work responsibilities as the reason for not regular utilizing GMP services. To address these barriers, we recommend implementing flexible GMP service hours at health facilities to accommodate mothers household schedules, integrating GMP services with other routine maternal and child health visits to reduce the number of separate visits required, establishing community-based GMP services closer to residential areas to minimize travel time, and engaging family members, particularly fathers, to share household responsibilities and support mothers in attending GMP sessions. The study emphasizes the need for strategies to enhance maternal knowledge, public awareness to increase the use of growth monitoring and promotion services. Additional studies are needed to understand the long-term effects of low GMP utilization on child health outcomes in Nepal. Future studies should adopt a broader perspective by incorporating a wider range of variables, including cultural beliefs, health system factors, socioeconomic determinants, paternal involvement, and community support systems, to provide a more comprehensive understanding of GMP utilization and its influencing factors. Extending the geographical scope might enhance generalizability, enable regional comparisons, ultimately informing more effective child health strategies, and improve child health outcomes.

## Data Availability

Data will be available upon reasonable request.

## Abbreviations

ANC: Antenatal care
AOR: Adjusted Odd Ratio
CI: Confidence Interval
NDHS: Nepal Demographic Health Survey
GM: Growth Monitoring
GMP: Growth Monitoring and Promotion
PNC: Postnatal Care
